# Validation of a Novel Smartphone-Based Point-of-Care Semen Analysis System to Evaluate Male Reproductive Potential: A Concordance Study with Computer-Assisted Sperm Analysis

**DOI:** 10.3390/diagnostics16111631

**Published:** 2026-05-26

**Authors:** Byeong Jun Mun, Seung A Oh, Jin Young An, Seong Jung Kim, Yu Ha Shim, Ji Soo Ryu, Hyun Seung Lee, Tae Eun Shin, Ji Hoon Kim, Yu Jin Lee, Jun Ho Ji, Dae Keun Kim, Jae Ho Lee

**Affiliations:** 1 Department of Biomedical Science, College of Life Science, CHA University, Pocheon 11160, Republic of Korea; bjmun@chauniv.ac.kr (B.J.M.); dhtmddk0102@gmail.com (S.A.O.); ajy6866@chauniv.ac.kr (J.Y.A.); hamin9247@gmail.com (S.J.K.); yuhaa525@gmail.com (Y.H.S.); jisuho5229@gmail.com (J.S.R.); 2 CHA Fertility Center, Seoul Station, Hangang-daero, Jung-gu, Seoul 04637, Republic of Korea; hs9052@chamc.co.kr (H.S.L.); s7530254@chamc.co.kr (T.E.S.); kdg070723@gmail.com (D.K.K.); 3 INTIN Inc., 52, Cheombok-ro, Dong-gu, Daegu 41068, Republic of Korea; ceo@intin.kr (J.H.K.); yj.lee@intin.kr (Y.J.L.); jh.ji@intin.kr (J.H.J.)

**Keywords:** male infertility, point-of-care testing, CASA, sperm motility, diagnostic accuracy

## Abstract

**Background/Objectives:** Male factor infertility contributes to approximately 40–50% of infertility cases globally, yet traditional laboratory-based semen analysis often imposes logistical and psychological barriers. This study aimed to evaluate the analytical performance and diagnostic concordance of a novel smartphone-based point-of-care testing (POCT) system, Hagobogo Pro, compared with a laboratory-based computer-assisted sperm analysis (CASA) reference system. **Methods:** This retrospective validation study analyzed 520 video microscopy clips obtained from 104 men undergoing infertility evaluation at a tertiary fertility center. Following World Health Organization (WHO) 2021 guidelines, sperm concentration and total motility were measured using the Hagobogo Pro smartphone device and the reference system. Analytical performance was assessed based on intra-assay precision, operational time, and method agreement using Passing–Bablok regression, Bland–Altman analysis, and Spearman correlation. **Results:** The smartphone-based system demonstrated strong analytical agreement with the CASA reference, with high correlations observed for sperm concentration (ρ = 0.943) and motility (ρ = 0.7335). Bland–Altman analysis indicated minimal systematic bias, and intra-assay precision showed coefficients of variation below 6%. There were no statistically significant differences in mean parameters between the smartphone device, CASA, and manual assessment. **Conclusions**: The Hagobogo Pro platform enables rapid, reliable, and standardized sperm concentration and motility quantification, and results showed good agreement with laboratory CASA. While not a replacement for holistic laboratory evaluations, this technology improves access to preliminary male fertility screening and may empower patients by mitigating barriers to initial testing.

## 1. Introduction

Male factor pathophysiology is a primary or contributory etiology in approximately 40% to 50% of all global infertility cases [1]. Consequently, precise, timely, and reproducible evaluation of male reproductive potential is a foundational diagnostic step in guiding infertile couples toward appropriate assisted reproductive technology (ART) interventions, including in vitro fertilization (IVF) and intracytoplasmic sperm injection (ICSI) [2]. Conventional semen analysis (CSA) remains the cornerstone of andrological evaluation, providing information on spermatozoa concentration, total and progressive motility, and strict morphological indices [3].

Despite its diagnostic importance and widespread clinical implementation, the traditional manual CSA process is encumbered by methodological and logistical limitations that compromise standardization and accessibility [4]. From a patient perspective, seminal sample collection in a clinical setting frequently induces psychological stress, anxiety, and embarrassment [5]. These psychophysiological stressors have been documented to alter seminal parameters and, more broadly, discourage patient participation in early fertility screenings [6]. Methodologically, manual microscopic assessment is subjective, labor-intensive, and reliant on the embryologist’s or laboratory technician’s expertise and technical proficiency [7]. This reliance on human judgment can lead to inter- and intra-operator variability, compromising the reproducibility and standardization mandated by World Health Organization (WHO) diagnostic guidelines [8].

To mitigate these analytical deficiencies, computer-assisted sperm analysis (CASA) systems were introduced into specialized clinical practice over four decades ago [9]. Modern, highly calibrated CASA platforms such as the Sperm Class Analyzer (SCA™) and the Hamilton Thorne IVOS system leverage advanced microscopic optics and algorithmic kinematic processing to automate spermatozoa parameter quantification, significantly enhancing diagnostic objectivity [10]. Nevertheless, while CASA systems have demonstrably elevated the standard of care in tertiary clinical settings, their high costs, operational complexity, and restriction to highly specialized andrology laboratories render them largely inaccessible for preliminary screening or decentralized point-of-care applications [11].

The recent COVID-19 pandemic catalyzed an unprecedented paradigm shift in global healthcare delivery, accelerating the adoption of telemedicine and remote, non-face-to-face diagnostic solutions across numerous medical disciplines, including reproductive endocrinology [12]. This shift has driven the development and commercialization of at-home direct-to-consumer (DTC) male infertility diagnostic kits. Early iterations of home-based solutions primarily included qualitative immunochromatographic assays (e.g., SpermCheck Fertility) and semi-quantitative microfluidic devices [13]. While these earlier kits offered enhanced patient privacy and cost-effectiveness, their clinical utility was limited by their qualitative nature [12].

These diagnostic tools leverage the global adoption of advanced smartphone technology. Novel smartphone-based semen analysis systems integrate high-resolution mobile cameras, micro-optical attachments, and automated image analysis software to execute fully automated quantitative analyses of kinematic sperm parameters. This technological convergence has the potential to bring objective, highly reproducible semen analysis directly to the patient, aligning with the WHO’s endorsement of computer-aided diagnostic systems.

Despite these promises, devices’ diagnostic accuracy and clinical reliability must be evaluated using gold standard clinical methodologies. Persistent questions remain regarding their capacity to accurately distinguish normozoospermic from oligozoospermic or asthenozoospermic samples based on established WHO diagnostic thresholds. Failure to empirically validate DTC kits’ inter-method precision could precipitate misdiagnoses, delaying ART interventions. Therefore, this study aimed to analyze the analytical efficiency, intra-assay precision, and diagnostic concordance of a newly engineered smartphone-based AI seminal diagnostic platform (Hagobogo Pro) against conventional manual assessment and laboratory-based CASA systems.

## 2. Materials and Methods

### 2.1. Study Design and Clinical Data Acquisition

This retrospective validation study used clinical andrological data obtained subsequent to routine IVF treatment cycles. The study protocol was reviewed and approved by the Institutional Research and Ethical Committees of Gangnam CHA Hospital (approval number: GCI 2025-08-008), Republic of Korea. All protocols adhered to the governing IRB’s ethical standards for the protection of patient health information.

The seminal sample repository comprised 520 digital video microscopy clips procured from 104 infertile male patients attending the CHA Fertility Center, Seoul Station. Prior to sample provision, participants were instructed to maintain a standard sexual abstinence interval of 2 to 3 days. Seminal fluid was collected via masturbation into sterile, non-toxic specimen containers and incubated at 37 °C for 30 min to facilitate physiological liquefaction. Seminal analyses were conducted in accordance with the standardized protocols delineated in the World Health Organization (WHO)’s 6th edition laboratory manual [8].

### 2.2. Reference Computer-Assisted Sperm Analysis (CASA)

Baseline seminal quantification was performed using a laboratory-based computer-assisted sperm analysis system, Sperm Class Analyzer® CASA System (SCA® software v6.5; Microptic S.L., Barcelona, Spain). The system digitally captured the kinematic trajectories of individual spermatozoa and categorized their motility phenotypes. A proprietary INTIN object detection computational model was deployed to isolate and trace spermatozoa spatial coordinates across successive video frames. These dynamic movement vectors were mathematically synthesized into composite trajectory schematics from which spermatozoa concentration and motility percentages were extrapolated.

### 2.3. Investigational Smartphone-Based Assay (Hagobogo Pro)

**Figure 1:**
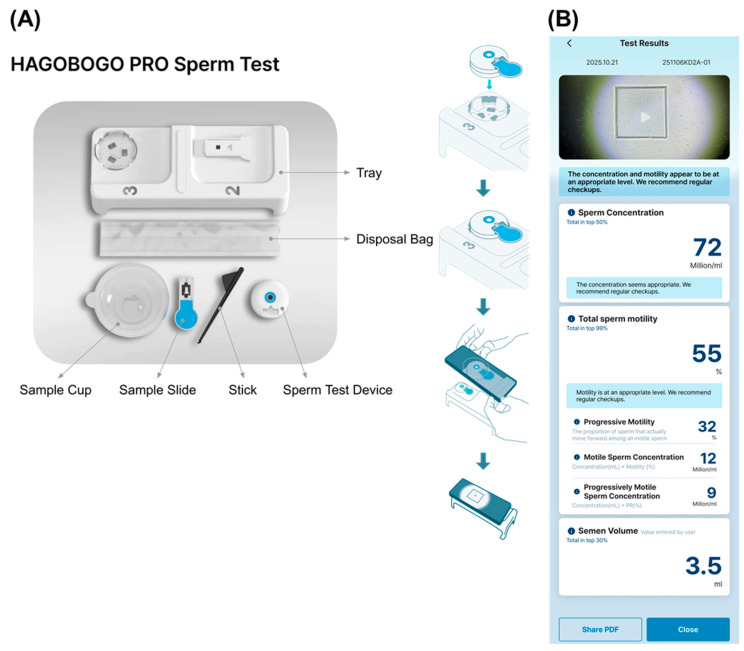
System architecture and operational workflow of the investigational smartphone-based point-of-care semen analysis device (Hagobogo Pro). (**A**) Schematic of the hardware: micro-chamber sample slide, central optical testing module, stabilization tray, and sample collection accessories. (**B**) Workflow illustrating sample loading, docking and alignment of smartphone optics, and the proprietary automated sperm detection software-driven mobile application interface. The digital display demonstrates the automated rendering of quantified seminal parameters—including spermatozoa concentration and motility fractions—against World Health Organization (WHO) diagnostic thresholds.

The investigational point-of-care device, Hagobogo Pro (INTIN Inc., Daegu, Republic of Korea), is a miniature optical microscopy peripheral designed to interface with standard smartphone camera arrays and a proprietary automated sperm detection software application for automated andrological analysis. The modular hardware architecture comprises four elements: a specialized micro-chamber sample slide, optical housing featuring a magnification lens, a stabilization cradle, and the integrated smartphone application (Figure 1A). The system uses the smartphone’s optics to capture high-magnification, high-frame-rate videos of the seminal aliquot.

A precisely measured aliquot of fully liquefied semen was pipetted into the dedicated testing chamber and hermetically sealed. The slide was docked into the optical housing, which was engaged with the stabilization cradle to enforce an immutable focal length. Upon optimal alignment of the smartphone’s optics, the application initiated video documentation. Following data capture, the automated sperm detection software processed the video matrix frame by frame (Figure 1B). The computational model identified distinct cellular morphologies to compute the absolute spermatozoa concentration (expressed in M/mL). The algorithm also evaluated each cell’s spatial displacement and velocity vectors to quantify the total motility (%). The manufacturer-reported analytical limits for the system are 0 to 208 million/mL for concentration and 0% to 100% for motility parameters. Quantitative metrics, juxtaposed against WHO 6th edition diagnostic thresholds, are rendered instantaneously on the user interface.

### 2.4. INTIN Detection Algorithm

The INTIN detection algorithm was developed using a proprietary image analysis approach to identify and quantify spermatozoa from video frames. The algorithm processes sequential frames to detect moving objects consistent with sperm characteristics and tracks their trajectories to estimate concentration and motility. To account for variations in imaging conditions across different smartphone devices, an adaptive parameter adjustment strategy was applied, enabling consistent detection performance under varying optical and environmental conditions. The algorithm was developed and evaluated using a dataset comprising 1,050,570 video clips, corresponding to approximately 368,921,312 extracted image frames. Validation was performed by comparing the algorithm-detected sperm count with manual counts conducted by experienced embryologists. Detected sperm were marked with bounding boxes, and expert reviewers independently verified spermatozoa presence and counts. The algorithm’s detection accuracy was approximately 90%, with a low false detection rate observed during expert validation.

### 2.5. Statistical Analysis and Diagnostic Concordance Evaluation

The device’s intra-assay precision was quantified by calculating the Coefficient of Variation (CV) across three independent repeated measurements of randomly selected seminal aliquots. Operational time efficiency was chronometered from the moment of sample introduction to the generation of the final digital report.

To assess methodological agreement and systematic bias, the quantitative outputs from the Hagobogo Pro platform were mapped against the laboratory CASA reference standard using Passing–Bablok regression analysis and Bland–Altman plots. Diagnostic concordance—specifically, the device’s ability to identify “normal” and “abnormal” samples based on WHO 6th edition lower reference limits (concentration: 16 M/mL; total motility: 42%)—was evaluated using Cohen’s kappa (κ). All statistical computations were executed using Python architecture (version 3.10.11; Python Software Foundation, Wilmington, DE, USA), with a *p*-value threshold of <0.05 indicating statistical significance.

## 3. Results

### 3.1. Baseline Clinical Characteristics of the Study Cohort

A total of 104 male patients undergoing clinical evaluation for subfertility were enrolled. Participants’ mean age was 40.36 ± 4.18 years (mean ± standard deviation (SD)).

Seminal parameters calculated by the reference CASA system are presented in Table 1. Mean seminal volume was 3.29 ± 1.6 mL, mean spermatozoa concentration was 82.97 ± 44.53 × 10^6^/mL, and mean total kinetic motility was 48.65 ± 16.46%. These baseline metrics, when compared with the WHO 2021 lower reference limits (1.4 mL for volume, 16 × 10^6^/mL for concentration, and 42% for motility), indicate predominantly normozoospermic mean values with substantial individual variance, reflecting a standard clinical infertility population.

To evaluate inter-methodological reliability, the mean concentration and motility values determined by the reference CASA system were cross-analyzed against outputs from the investigational INTIN (Hagobogo Pro) device and standard manual microscopic assessments performed by embryologists using a Makler counting chamber (Table 2). The results for spermatozoa concentration were highly homogenous: 82.97 ± 44.53 × 10^6^/mL (CASA), 79.55 ± 38.95 × 10^6^/mL (INTIN), and 79.91 ± 41.49 × 10^6^/mL (embryologist assessment). Total motility metrics were also similar: 48.65 ± 16.46% (CASA), 45.80 ± 15.53% (INTIN), and 46.58 ± 12.94% (embryologist). There was no statistically significant difference across the three diagnostic methodologies (*p* > 0.05), validating that data extrapolated from the decentralized INTIN platform are comparable with laboratory metrics.

### 3.2. Diagnostic Concordance and Methodological Agreement

**Figure 2:**
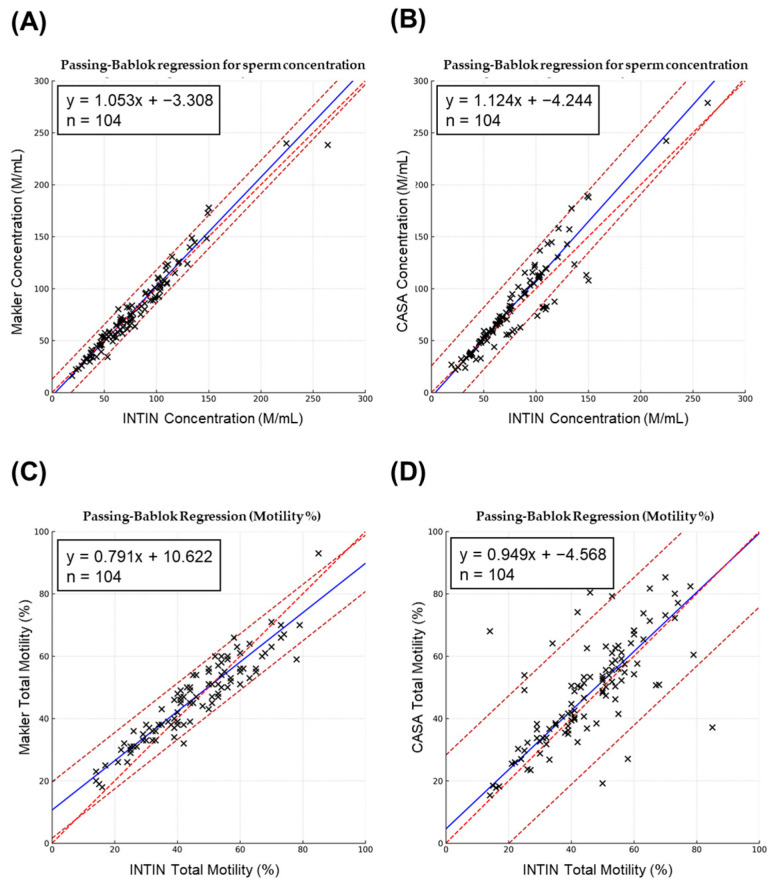
Evaluation of the automated sperm detection software-augmented smartphone assay (INTIN/Hagobogo Pro) using Passing–Bablok regression analysis. The scatter plots (left panels) illustrate the linear relationship and structural agreement between the device and established clinical reference standards in a cohort of 104 subfertile men. In each panel, the blue solid line represents the Passing-Bablok regression line, the red dashed diagonal line indicates the identity line (y = x), and the dark red dashed lines represent the 95% confidence interval of the Passing-Bablok regression line. (**A**) Correlation of sperm concentration (M/mL) between the smartphone assay and manual Makler chamber assessment performed by senior embryologists. (**B**) Correlation of sperm concentration (M/mL) between the smartphone assay and the reference computer-assisted sperm analysis (CASA) system. (**C**) Agreement of total motility (%) between the smartphone assay and manual Makler chamber assessment. (**D**) Agreement of total motility (%) between the smartphone assay and the reference CASA system.

To further elucidate the analytical concordance, the Hagobogo Pro system was subjected to Passing–Bablok regression and Spearman correlation modeling against manual expert evaluation and CASA (Figure 2). For spermatozoa concentration, Passing–Bablok regression comparing the investigational device against expert embryologist assessment (Makler chamber) demonstrated a strong linear correlation (y = 1.053x − 3.308; Spearman’s ρ = 0.95, *p* < 0.001) (Figure 2A). When compared to the CASA reference, the device maintained an exceptionally high correlative index and linear agreement (y = 1.124x − 4.244; ρ = 0.943, *p* < 1.24 × 10^−50^), corroborating the device’s reliability (Figure 2B). Evaluation of total kinetic motility yielded similarly robust data. Regression analysis underscored a high degree of clinical concordance between the smartphone assay and expert manual assessment (y = 0.791x + 10.622; ρ = 0.94, *p* < 0.001) (Figure 2C). Comparing the investigational device and the CASA platform generated a moderate to strong diagnostic correlation and excellent linear fit (y = 0.949x + 4.568; ρ = 0.7335, *p* < 8.33 × 10^−19^), further substantiating the algorithm’s computational robustness (Figure 2D).

To further evaluate the diagnostic agreement, samples were classified based on the WHO 2021 threshold for total motility (≥42%). In Table 3, Cohen’s kappa analysis demonstrates substantial agreement between INTIN and CASA (κ = 0.680). Agreement between INTIN and expert assessment (Makler chamber) was higher (κ = 0.817), indicating almost perfect agreement, while CASA and expert assessment also showed substantial agreement (κ = 0.777).

### 3.3. Diagnostic Concordance and Systematic Bias

**Figure 3:**
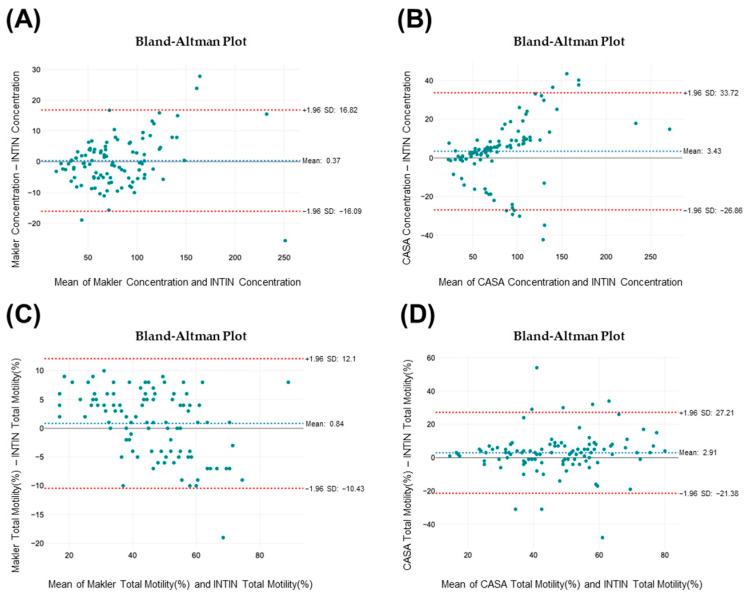
Assessment of diagnostic concordance and systematic bias of the automated sperm detection software-augmented smartphone assay (INTIN/Hagobogo Pro) using Bland–Altman analysis. The difference plots (right panels) quantify the proportional bias and 95% limits of agreement (LoA) between the device and reference standards. The central horizontal line represents the mean bias, with shaded areas or dashed lines indicating the 95% LoA (±1.96 SD). (**A**) Concentration vs. manual assessment: Evaluation of agreement for spermatozoa concentration (M/mL) between the smartphone assay and manual Makler chamber evaluation. (**B**) Motility vs. manual assessment: Difference plot showing the agreement of total kinetic motility (%) between the investigational device and expert manual assessment. (**C**) Concentration vs. CASA: Systemic bias evaluation for spermatozoa concentration comparing the point-of-care device to the gold standard CASA system. (**D**) Motility vs. CASA: Evaluation of agreement for total motility metrics between the device and the reference CASA platform.

To assess potential bias, Bland–Altman analyses were performed to compare the smartphone assay against expert manual assessment and the CASA system (Figure 3). For spermatozoa concentration, the difference plot revealed a minimal mean bias of 0.37 M/mL between the smartphone assay and manual Makler chamber counts, with 95% limits of agreement (LoA) ranging from −16.09 to 16.82 M/mL (Figure 3A). When compared with the CASA platform, the analysis indicated a slightly higher mean difference of 3.43 M/mL (95% LoA: −26.86 to 33.72 M/mL) (Figure 3B). The comparison against CASA demonstrated some proportional bias, with the difference between the two methods increasing at higher mean sperm concentrations. Diagnostic agreement for total kinetic motility metrics was similarly evaluated. The point-of-care device demonstrated a mean bias of only 0.84% compared with expert manual assessment, with a narrow 95% LoA between −10.43% and 12.1% (Figure 3C). Against the CASA platform, the smartphone assay yielded a mean bias of 2.91%, with the 95% LoA spanning from −21.38% to 27.21% (Figure 3D). These results demonstrate that the smartphone-based platform achieves analytical accuracy and diagnostic parity comparable to specialized CASA and highly trained clinical personnel, maintaining statistical deviations well within acceptable clinical tolerance.

## 4. Discussion

The epidemiological burden of male factor infertility—accounting for an estimated 40% to 50% of global infertility cases—necessitates continuous optimization of andrological diagnostic pathways. While conventional laboratory-based semen analysis remains the diagnostic gold standard, its routine clinical application is intrinsically hindered by significant logistical barriers, psychological burden on the patient, and the necessity for highly specialized laboratory infrastructure to mitigate the known intra-observer variability associated with manual evaluation. These well-documented clinical bottlenecks emphasize the need for more accessible, standardized, and patient-centered diagnostic solutions.

In this evolving clinical landscape, advanced point-of-care and home-based semen analysis technologies represent a promising adjunct to conventional diagnostic pathways, addressing key limitations of traditional methodologies. While early iterations of DTC testing were confined to rudimentary qualitative outputs (e.g., SpermCheck), the integration of advanced optics and computational algorithms into consumer smartphones—as demonstrated by platforms like the LensHooke^®^ X1 PRO and the Hagobogo Pro evaluated herein—has substantially improved the feasibility of quantitative remote semen analysis. The data presented in this validation study suggest that these automated sperm detection software-augmented POCT devices can yield analytical outputs that are generally concordant with laboratory CASA systems, enabling accurate stratification and potentially circumventing the psychological burden that frequently delays male fertility consultations.

Nevertheless, critical clinical caveats must be applied to the interpretation and widespread deployment of current home-based analytical platforms. Primarily, the limited scope of our clinical cohort (n = 104) and the retrospective nature of the analysis mandate subsequent, large-scale prospective validation trials across diverse demographic populations to establish generalizability more clearly. Furthermore, a significant limitation of contemporary POCT devices, including the investigational model, is their inability to execute comprehensive diagnostic evaluations; they currently lack the capacity to assess strict spermatozoa morphology, cellular vitality, or highly predictive functional molecular parameters, such as DNA fragmentation indices or oxidative stress markers [14,15]. Additionally, while the autonomy of home testing improves patient compliance, the variance in sample collection protocols initiated by untrained lay users introduces an unquantified pre-analytical variable that may impact clinical reliability. These factors underscore that while DTC devices may serve as useful preliminary screening tools, an over-reliance on their outputs must not preclude comprehensive clinical andrological evaluation for patients pursuing ART [13].

Looking toward the future of reproductive medicine, the continuous miniaturization of diagnostic technology may help address some of these current limitations. The eventual integration of sophisticated molecular and genetic diagnostics—capable of identifying complex epigenetic dysregulations, chromosomal aneuploidies, or aberrant microRNA biomarker expressions—into point-of-care microfluidic platforms may improve our understanding of idiopathic male infertility. Parallel advancements in machine learning and automated sperm detection software may also permit automated, more detailed morphological assessments via smartphone optics, facilitating personalized therapeutic algorithms directly from the initial patient interaction [16,17].

## 5. Conclusions

This comparative study demonstrates the clinical utility of the Hagobogo Pro smartphone-based platform. By enabling rapid, highly accurate, and standardized quantification of spermatozoa concentration and motility, this technology provides greater access to baseline andrological screening. While such devices cannot supplant the need for holistic, specialized laboratory evaluations, they represent an invaluable first-line diagnostic modality. By mitigating psychological and logistical barriers to initial testing, automated software-driven sperm detection technologies empower patients, expedite clinical interventions, and contribute to optimized prognostic outcomes for subfertile couples navigating the complexities of reproductive medicine.

## Figures and Tables

**Table 1:** Main seminal parameters quantified by the reference computer-assisted sperm analysis (CASA) system.

Parameter	Volume (mL)	Concentration (×10^6^/mL)	Motility (%)
WHO LRLs	1.4	16	42
CASA	3.29 ± 1.6	82.97 ± 44.53	48.65 ± 16.46

**Table 2:** Comparison of sperm concentration and motility across CASA, INTIN, and Makler chamber.

Parameter	CASA	INTIN	Makler Chamber	*p*-Value
Concentration (×10^6^/mL)	82.97 ± 44.53	79.55 ± 38.95	79.91 ± 41.49	ns
Motility (%)	48.65 ± 16.46	45.80 ± 15.53	46.58 ± 12.94	ns

Data are presented as mean ± standard deviation. ns, not significant (*p* ≥ 0.05).

**Table 3:** Diagnostic Agreement Analysis (WHO 2021 Threshold) [8].

Comparison Pair	Kappa Coefficient (k)	Strength of Agreement
INTIN vs. Expert Assessment	0.817	Almost Perfect
CASA vs. Expert Assessment	0.777	Substantial
INTIN vs. CASA	0.680	Substantial

## Data Availability

The raw data supporting the conclusions of this article will be made available by the authors upon request.
